# Escalation with Overdose Control Using Time to Toxicity for Cancer Phase I Clinical Trials

**DOI:** 10.1371/journal.pone.0093070

**Published:** 2014-03-24

**Authors:** Mourad Tighiouart, Yuan Liu, André Rogatko

**Affiliations:** 1 Samuel Oschin Comprehensive Cancer Institute, Biostatistics and Bioinformatics Research Center, Los Angeles, California, United States of America; 2 Winship Cancer Institute, Emory University, Atlanta, Georgia, United States of America; The James Cook University Hospital, United Kingdom

## Abstract

Escalation with overdose control (EWOC) is a Bayesian adaptive phase I clinical trial design that produces consistent sequences of doses while controlling the probability that patients are overdosed. However, this design does not take explicitly into account the time it takes for a patient to exhibit dose limiting toxicity (DLT) since the occurrence of DLT is ascertained within a predetermined window of time. Models to estimate the Maximum Tolerated Dose (MTD) that use the exact time when the DLT occurs are expected to be more precise than those where the variable of interest is categorized as presence or absence of DLT, given that information is lost in the process of categorization of the variable. We develop a class of parametric models for time to toxicity data in order to estimate the MTD efficiently, and present extensive simulations showing that the method has good design operating characteristics relative to the original EWOC and a version of time to event EWOC (TITE-EWOC) which allocates weights to account for the time it takes for a patient to exhibit DLT. The methodology is exemplified by a cancer phase I clinical trial we designed in order to estimate the MTD of Veliparib (ABT-888) in combination with fixed doses of gemcitabine and intensity modulated radiation therapy in patients with locally advanced, un-resectable pancreatic cancer.

## Introduction

Cancer phase I clinical trials constitute the first step in investigating the safety of potentially promising new cytotoxic or biological drugs in humans. In these studies, patients are accrued to the trial sequentially and the dose allocated to the next patient depends on the doses and dose limiting toxicity (DLT) status of all previously treated patients. The goal is to estimate a dose level that is associated with a pre-determined level of DLT.

Dose assignment is carried out after the DLT status of patients under observation is resolved. This occurs within one cycle of therapy, which typically lasts 3 to 6 weeks. The target dose *γ* is referred to as the maximum tolerated dose (MTD), and is defined as the dose that is expected to produce DLT in a specified proportion *θ* of patients:
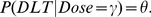
(1.1)


Several statistical methodologies have been proposed in the literature to select the MTD, see [1], [2], [3], [4] for a review. An important class of methods that produce consistent sequences of doses are Bayesian adaptive designs, such as the continual reassessment method (CRM) proposed by O'Quigley et al. [5] and its modifications [6], [7], [8], [9], [10], and the escalation with overdose control (EWOC) method described by Babb et al. [11], Zacks et al. [12], Tighiouart et al. [13], [14], [15], [16], and Tighiouart and Rogatko [17].

A limitation with this design is that the toxicity outcome is coded as a binary variable, presence or absence of DLT. For trials where the length of a cycle of therapy is two months or longer as in treatments involving radiotherapy, total trial duration can be very long making the trial practically non-feasible. This limitation motivated the development of models to estimate the MTD that take into account the amount of time patients are under observation when new patients are about to enter the trial. Cheung and Chappell [18] extended the CRM to allow late-onset toxicity. Their approach was to allocate weights to account for the time it takes for a patient to exhibit DLT. A similar approach was adapted to EWOC by Mauguen et al. [19]. They showed that the design operating characteristics of EWOC in terms of safety and MTD recommendation were maintained while the length of the trial was reduced when compared with EWOC. These approaches assume that the weights are linear function of the time to follow up with value equal to 1 if a patient experiences DLT. This implies that patients who experience DLT at *different* time points will contribute the *same* information to the likelihood function.

In this paper, we develop a class of adaptive Bayesian models that take into account not only the status of DLT during the observation window, but also the time it takes for the patient to exhibit DLT. These designs are expected to be more efficient when estimating the MTD since more information is being collected and used in the trial. Design operating characteristics are studied using extensive simulations and are compared to two versions of EWOC and time to event EWOC (TITE-EWOC) described in [19].

## Methods

In this section, we describe our design by assuming that the risk of DLT given dose follows a proportional hazards model [20]. The design is termed EWOC-PH.

### 2.1 EWOC-PH

Let *T*_1_, *T*_2_,…, *T_n_* be nonnegative absolutely continuous random variables representing time to DLT. Suppose that each patient is observed up to time *τ* after he or she is given the treatment. In practice, *τ* is usually equal to one cycle of therapy, equivalent to 3 or 4 weeks since drug administration or even longer for therapies involving radiation. Let *D_n_*  =  {(*Y_i_*, *x_i_*, *δ_i_*), *i* = 1,…,*n*} be the observed data, where *Y_i_*  =  min(*T_i_*, *τ*), *x_i_* is the dose allocated to patient *i*, and *δ_i_* = *I*(*T_i_*≤*τ*). In other words, if *δ_i_* = 1, we observe a DLT within the observation window, otherwise, the time to DLT is censored at time *τ*. Note that here, we are assuming that the DLT status of all patients can be resolved by the end of the cycle of therapy. The methodology is equally applicable to the case where censoring occurs before time *τ*. For instance, the time to DLT is censored before time *τ* if a patient is withdrawn from the study due to disease progression or if a patient exhibits a severe adverse event not attributed to the treatment. Following the classical definition of the MTD where the DLT outcome variable is binary given in (1.1), we define the MTD *γ* as the dose at which a proportion *θ* of patients exhibit DLT during the observation window [0, *τ*], i.e.
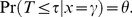
(2.1)


The value chosen for the target probability *θ* depends on the nature and clinical manageability of the DLT; it is set relatively high when the DLT is a transient, correctable or non-fatal condition, and low when it is lethal or life threatening. Suppose that dose levels in the trial are selected in the interval [*X*_min_, *X*_max_].

#### 2.1.1. Likelihood

We model the risk of DLT given dose *h*(*t*|*x*) by assuming that patients given different doses of an agent have proportional risks of DLT. Following Cox proportional hazards model [20], we have 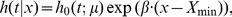
(2.2)where *h*_0_(*t*; *µ*) is the baseline hazard function corresponding to the risk of DLT for a patient given dose *X*_min_ and *µ* is a vector of parameters associated with the parametric baseline hazard. The regression effect 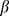 represents the fixed dose effect on the risk of DLT. We assume that 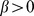 so that the hazard of DLT is an increasing function of dose. After enrolling 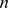 patients in the trial, the likelihood function for the parameters is
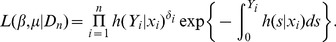
(2.3)


We reparameterize model (2.2) in terms of *γ* and *ρ*_0_, the probability that a DLT manifests within the first cycle of therapy for a patient given dose *x* = *X*_min_. This reparameterization is convenient to clinicians since *γ* is the parameter of interest and prior information on *ρ*_0_ may be available from other trials or from trials using similar agents. Since there is a one to one correspondence between the survival function and hazard function 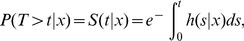
(2.4)it follows that
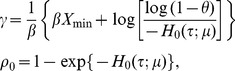
(2.5)where 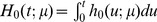 is the cumulative baseline hazard function.

Assuming that the baseline instantaneous risk of DLT follows an exponential distribution with hazard function *h*_0_(*t*;*µ*)  =  *µ*, one can show that 
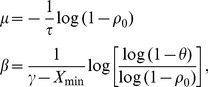
(2.6)and the likelihood (2.3) becomes
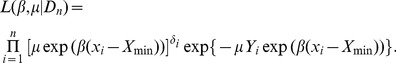
(2.7)


Other flexible parametric baseline hazards such as Weibull and lognormal distributions can be used at the expense of increased number of parameters. Using (2.6) and (2.7), one can easily write the likelihood of the reparameterized model *L*(*ρ*_0_,*γ|D_n_*). From (2.6), the assumption *β*>0 implies that 0<*ρ*_0_<*θ*.

#### 2.1.2. Prior and Posterior Distributions

Let *g*(*ρ*_0_,*γ*) be a prior distribution on *ρ*_0_ and *γ* on [0, *θ*]×[*X*_min_, *X*_max_]. Using Bayes rule, the posterior distribution of the model parameters is proportional to the product of the likelihood and prior distribution
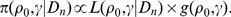
(2.8)


We designed an MCMC sampler based on the Metropolis-Hastings algorithm [21], [22] to obtain model operating characteristics. We also used WinBUGS [23] to estimate features of the posterior distribution of the MTD and design a trial. In the absence of prior information about the MTD and probability of DLT at *X*_min_, independent vague priors are selected for *ρ*_0_ and *γ*.

#### 2.1.3 Trial Design

Dose levels in the trial are selected in the interval [*X*_min_, *X*_max_]. The adaptive design proceeds as follows. The first patient receives the dose *x*_1_ = *X*_min_. If this patient experiences DLT within the observation window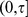, then we would recommend stopping the trial. Otherwise, the marginal posterior cdf of the MTD given that the first patient did not exhibit DLT by the end of the cycle of therapy is denoted by Π_1_(*γ*)  =  Π(*γ* | (*τ*, *x*_1_, 0)). The second patient receives the dose 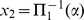 so that the posterior probability of exceeding the MTD is equal to the feasibility bound *α*. This is the overdose protection property of EWOC, where at each stage of the design, we seek a dose to allocate to the next patient while controlling the posterior probability of exposing patients to toxic dose levels. Suppose the *k-*th patient is ready to enter the trial at time *t_k_*. We then calculate 

Π*_k_*_-1_(*γ*)  =  Π(*γ* | (*Y_i_*, *x_i_*, *δ_i_*), *i* = 1,…,*k*-1) up to time *t_k_*. Note that here, *Y_i_* is either equal to *τ* if patient *i* already finished one cycle of therapy with no evidence of DLT by the time patient *k* is ready to enter the trial, or *Y_i_* is the time since patient *i* was given dose *x_i_* until time *t_k_* if this patient is still at risk by this time. Otherwise, *Y_i_* is the time to DLT for that patient. The *k-*th patient receives the dose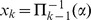. The trial proceeds until a pre-determined number of patients are enrolled to the trial. At the end of the trial, we estimate the MTD as the median of the posterior distribution of *γ*. We note that here, information on the DLT status of a patient past the observation window 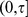 is not used in the model due to the definition of the MTD in (2.1). Such data may be appropriate to use if one is interested in optimizing both dose and number of cycles as in Braun et al. [24].

### 2.2 TITE-EWOC

Cheung and Chappell [18] extended the CRM to allow patients to enter the trial continuously, and called the design time to event CRM, TITE-CRM. Their approach was to allocate weights to take into account the time it takes for a patient to exhibit DLT. The method was further studied by Polley [25] to accommodate situations where we have fast patient accrual. TITE-CRM was adapted to EWOC by Mauguen et al. [19]. They assumed that the probability of DLT is given by 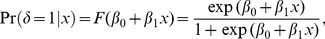
(2.9)where *β*_1_>0. Letting *w_i_* = *Y_i_*/*τ* if *δ_i_* = 0 and *w_i_* = 1 if *δ_i_* = 1, the corresponding likelihood function is
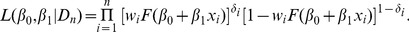
(2.10)


The model is further reparameterized in terms of *ρ_0_* and *γ* as in Section 2.1.1. These parameters are given by:
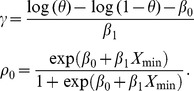
(2.11)


We note that this approach implies that patients who experience DLT at *different* time points will contribute the *same* information to the likelihood function. Trial design proceeds as described in Section 2.1.3.

### 2.3 Characteristics of EWOC-PH

The proposed design EWOC-PH assigns dose levels to future patients by taking into account the most recent DLT status of currently and previously treated patients according to the following properties.

1. At each stage of the design, we seek a dose to allocate to the next patient while controlling the posterior probability of exposing patients to toxic dose levels.
2. Suppose the DLT status of the first *k*-2 patients has been resolved. If patient *k*-1 does no exhibit DLT by the time patient *k* is ready to be enrolled to the trial at time *t_k_*, then the longer the time *t_k_*, the higher the recommended dose for patient *k* is.
3. Suppose the DLT status of the first *k*-2 patients has been resolved. If patient *k*-1 exhibits DLT shortly after he or she is given dose *x_k_*_-1_, then the dose recommended for the next patient is much lower than the dose given to patient *k* had patient *k*-1 exhibited DLT later in the cycle.

Property (i) is the overdose protection defining characteristic of EWOC which is also satisfied by TITE-EWOC but not by TITE-CRM. Property (ii) is intuitively appealing and although not mentioned in [18] and [19], it is shared by both TITE-CRM and TITE-EWOC. In fact, the property holds because the weight function *w* in (2.10) is an increasing function of *Y* as is shown in the proof of Theorem 1 below. Property (iii) is also naturally appealing because the amount of dose level reduction is a decreasing function of the time it takes for a patient to exhibit DLT. Property (iii) does not hold for TITE-CRM and TITE-EWOC since patients who exhibit DLT at different time points contribute the same weight in the likelihood function (2.10). Characteristics (ii) and (iii) are summarized in the following theorem.

THEOREM 1. *Let D_k_  = {(Y_1_, x_1_, δ_1_),…,(Y_k_, x_k_, δ_k_)} be the data on the first k patients generated by the design described in Section 2.1.3 and Π_k_(γ;Y_k_) be the cdf of γ given the data D_k_. Let 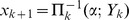 and 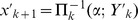. Suppose that for all i = 1,…,k-1, either δ_i_ = 1 or (Y_i_, δ_i_)  =  (τ, 0). Then, 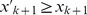 whenever 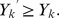 Furthermore, if the data D_k_ is generated by TITE-EWOC in Section 2.2 or TITE-CRM, and if δ_k_ = 0, then 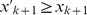 whenever 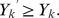
*

*Proof.*

Let
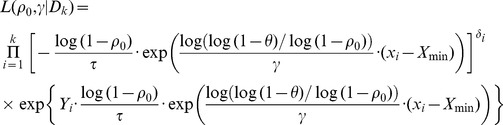



be the likelihood (2.7) reparameterized in terms of *ρ*_0_ and *γ*. To simplify notation and presentation of the proof, we assume that *X*_min_ = 0, *X*_min_ = 1, *τ* = 1, and *ρ*_0_ is fixed. Let *L_k_*(*γ*)  =  *L_k_*(*ρ*_0_,*γ*|*D_k_*), *π*(*γ*) be a proper prior density for *γ*, and 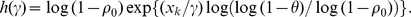 We note that since 

0<*ρ*_0_<θ, the function *h*(•) is negative and monotonically increasing in *γ.* Using Bayes' rule, the posterior c.d.f Π*_k_*(*t*;*Y_k_*) of the MTD *γ* is 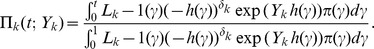



It follows that



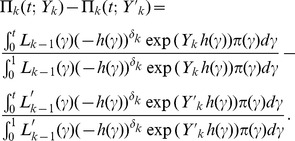
Since we are assuming that the DLT status of the first *k*-1 patients has been resolved, i.e., either *δ_i_ = *1 or (*Y_i_, δ_i_*) * = * (*τ,* 0) for *i≤k-*1, then 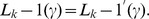 Hence, 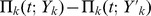


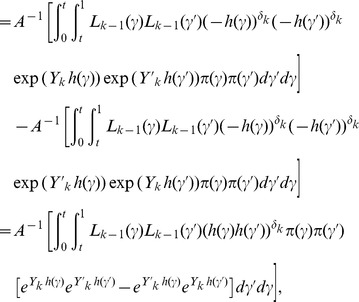
where
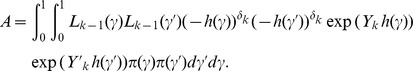



Since 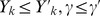 and *h*(.) is increasing in *γ*, then 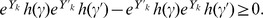 Furthermore, since *h*(·) is negative, *h*(*γ*)*·h*(*γ*′) is nonnegative. Hence, 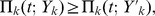 which implies that 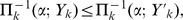 that is 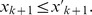 This completes the proof of the first part of Theorem 1.

If *δ_k_* = 0, then the likelihood (2.10) for TITE-EWOC is *L_k_*(*γ*)  =  *L_k-1_*(*γ*)(1-*w_k_*(*Y_k_*) *F*(*γ*;*x_k_*)), where *w_k_*(*Y_k_*) is the weight function defined in Section 2.2 and *F*(*γ*;*x_k_*) is the logistic function in (2.9) reparameterized in terms of the MTD *γ*. Using similar calculations as above, we have
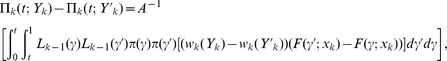
where
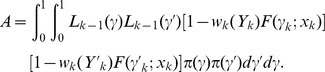



Since *w_k_*(*Y_k_*) is monotonically increasing in *Y_k_* and assuming that *F*(*γ*;*x_k_*) is decreasing in *γ*, which is the case for the logistic function, then 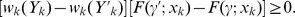 Hence, 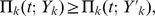 which implies that 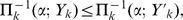 that is 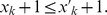 A similar argument shows that the property holds for the TITE-CRM.

### 2.4 Coherence of EWOC-PH

In the design of Cancer phase I trials using cytotoxic agents, it is ethical not to escalate the current dose *x_k_* if patient *k* (currently treated at this dose level) exhibits DLT. Similarly, if patient *k* does not experience DLT by the end of the first cycle of therapy, then the dose recommended for patient *k*+1 should not be lower than *x_k_*. This property is known as Coherence and was introduced by Cheung [26]. The CRM as proposed in [5] was shown to be coherent by Cheung [26] and the coherence of EWOC was established by Tighiouart and Rogatko [17]. For time to event toxicity Bayesian adaptive models, the definition of coherence has been extended in [26] who also showed that TITE-CRM is coherent. However, coherence in escalation does not have a practical interpretation in the case of delayed toxicities. Following the definition of coherence for time to event DLT in [26], one can easily show that EWOC-PH is also coherent.

We note that here, properties (ii) and (iii) of Theorem 1 are different from the notion of coherence. Theorem 1 makes a statement about the dose to be given to patient *k* given the length of time patient *k*-1 is under observation; the longer the time patient *k*-1 is under observation with no evidence of DLT, the higher the dose to be allocated to patient *k*. A similar statement is made if the patient exhibits DLT. Unlike the notion of coherence, we are not comparing the doses of patient *k*-1 and patient *k*.

## Simulation Studies

### 3.1 Design Operating Characteristics

In the simulation studies, we compare the operating characteristics of EWOC-PH with the original EWOC, TITE-EWOC and EWOC-W. The original EWOC introduced by Babb et al. [11] assumes that the DLT outcome is binary and dose allocation is carried whenever a patient is available for treatment. It is not necessary to wait for the DLT status of patients under observation to be resolved. EWOC-W, which stands for EWOC “waiting” works just like EWOC except that patients are enrolled to the trial only after the DLT status of all previously treated patients have been resolved. Both EWOC and EWOC-W use a logistic model (2.9). The designs are compared with respect to safety of the trial and efficiency of the estimate of the MTD by simulating *m* = 1000 trials of *n* = 48 patients each. Specifically, we calculated the average bias 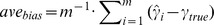, where 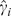 is the estimate of the MTD for the *i*-th trial and *γ_true_* is the true MTD under a particular scenario, the mean square error 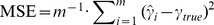, the average proportion of patients exhibiting DLT 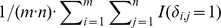, the percent of trials with estimated MTD within 10% of the dose range of the true MTD, and the percent of trials with DLT rate exceeding 40%. These last two summary statistics approximate the probability that a given trial will result in an estimated MTD close to the true MTD and the probability that a trial will be safe, respectively.

Dose levels have been standardized so that *X*_min_ = 0 and *X*_max_ = 1. We took *τ* = 1, the target probability of DLT was fixed at *θ* = 0.33, and the feasibility bound was set to *α* = 0.25. Independent uniform prior distributions were selected for *ρ*_0_ and *γ*, 

(*ρ*_0_, *γ*) ∼ Uniform([0, *θ*]×[0, 1]). We considered nine scenarios corresponding to three values for the true MTD *γ* = 0.3, 0.5, 0.7, three values for the accrual rate *ν* = 1, 2, and 4 patients per unit of time equal to the length of the observation window [0, *τ*], and fixed value of *ρ*_0_ = 0.05. In order to make a fair comparison between the different models and assess the performance of EWOC-PH under model misspecification, we simulated the times to DLT using two different models as described in the next section. For each scenario, patients enter to the trial according to a time homogeneous Poisson process with rate *ν*.

### 3.2 Models to generate time to DLT

The first model we considered for generating the time to DLT is similar to the proportional hazards model (2.2) but with a Weibull baseline hazard function
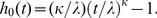
(3.1)


**Figure 1:**
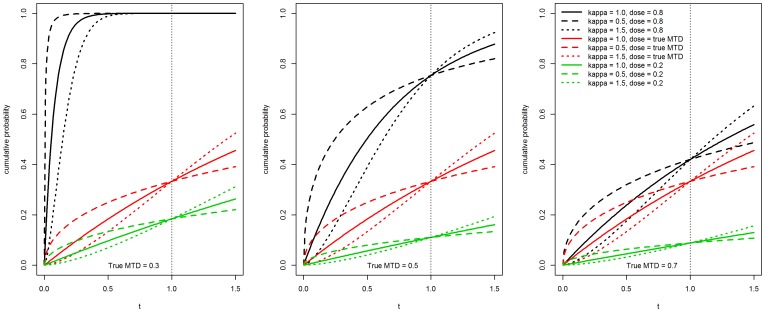
Cumulative distribution function plots for different values of *κ* and the true value of the MTD *γ* for various dose levels.

We took *κ* = 0.5, 1, 1.5. Note that the case *κ* = 1 corresponds to the exponential true model for EWOC-PH. Figure 1 shows the corresponding cdfs *P* (*T*≤*t* | *λ*, *κ*, *β*, dose  = *x*) for various values of the true MTD *γ*_true_, *γ*_true_ = 0.3, 0.5, 0.7 given three different doses *x* = 0.2, *x* = *γ*_true_, *x* = 0.8. The solid line corresponds to the true model EWOC-PH and serves as a reference for departure of the other cdfs from the true model. Note that these curves have been chosen so that they have the same MTD value in each scenario. This is accomplished by setting *P* (*T*≤*τ* | *λ*, *κ*, *β*, dose  = *γ*)  = *θ* and *P* (*T*≤*τ* | *λ*, *κ*, *β*, dose  =  *X*_min_)  =  *ρ*_0_. It then follows that *λ*  =  [−*κ*/((*κ*+1) log(1−*ρ*_0_))]^1/*κ*^ and *β*  =  *γ* ^−1^ log[−*κ* ^−1^ (*κ*+1) *λ^κ^* log(1−*θ*)].

The second model we considered is a non-proportional hazards model
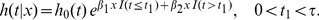
(3.2)


**Figure 2:**
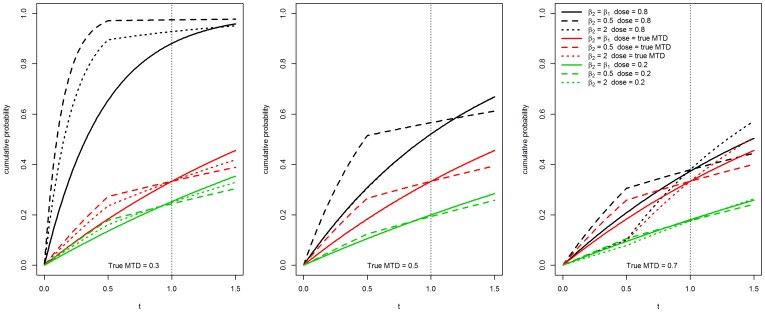
Cumulative distribution function plots for the non-proportional hazards model for different values of the true MTD *γ* and for various dose levels.

We used *h*_0_(*t*)  =  *b* = 0.15, *t*_1_ = 0.5, and two different values for *β*_2_, *β*_2_ = 0.5, 2. The values for *β*_1_ were selected to match the MTDs with the other models as above. It can be shown that *β*_1_  =  *γ* ^−1^ log[−*e^γβ^* ^2^ −2*b* ^−1^ log(1−*θ*)]. The corresponding cdfs are shown in Figure 2 along with the cdf of the true model which corresponds to the case *β*_1_ = *β*_2_. These models yield reasonable separations of the corresponding cdfs of the time to DLT from the true model and the extent of this separation increases with dose.

For each of the above models, let *T_i_* be the time to DLT for patient *i* generated from that model under a particular scenario. If *T_i_*>*τ*, then the observed time to DLT is censored at *τ* and the DLT response for EWOC and EWOC-W models is recorded as *δ_i_* = 0. Otherwise, *δ_i_* = 1 for EWOC and EWOC-W models.

## Results

### 4.1 Trial Duration

Table 1 shows the median trial duration across the *m* = 1000 simulated trials along with the first and third quartiles for the different designs as a function of the accrual rate. As expected, design of cancer phase I trials where the treatment is delayed until we observed the DLT status of all patients under observation can take at least twice as long when the expected number of available patients per cycle is 2, and can be more than four times longer when the accrual rate is 4.

**Table 1:** Median duration of the four trial designs by accrual rate.

Design	Accrual Rate		
	1	2	4
EWOC	48.2(44, 52.9)	25.2(22.8, 27.5)	13.8(12.8, 14.9)
EWOC-W	86.2(81.6, 90.7)	64.5(61.9, 67.6)	61.6(59.8, 63)
EWOC-PH	48.3(44.4, 53)	25.2(22.9, 27.5)	13.7(12.7, 15)
TITE-EWOC	48.4(44.2, 52.9)	25.3(23.2, 27.7)	13.7(12.7, 14.8)

The first and third quartiles are shown in parentheses.

### 4.2 Trial Efficiency

**Figure 3:**
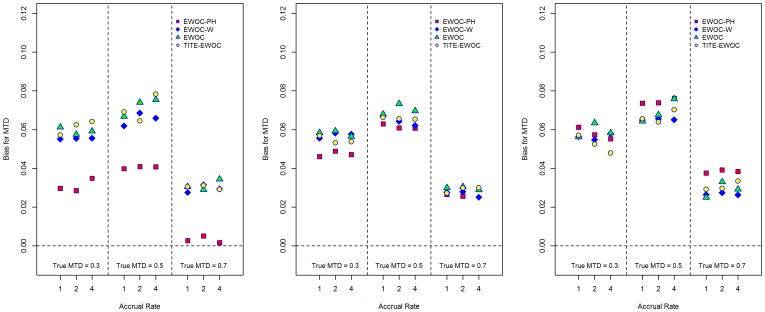
Average bias of the estimated MTD for each of the four models and nine scenarios when the time to DLT is generated from a proportional hazards model with Weibull baseline hazard rate with parameters *κ* = 0.5 (left panel), λ = *κ* = 1.0 (middle panel), and *κ* = 1.5 (right panel).

**Figure 4:**
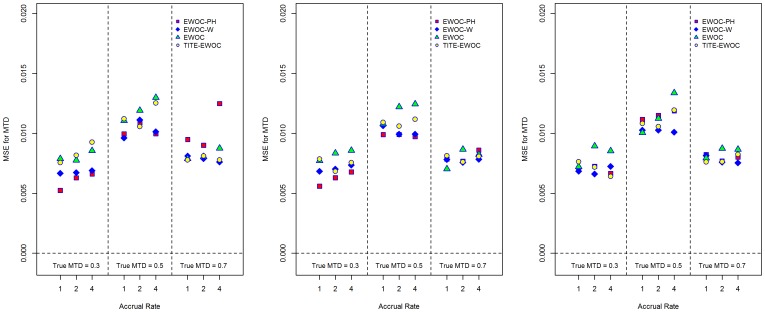
Mean square error of the estimated MTD for each of the four models and nine scenarios when the time to DLT is generated from a proportional hazards model with Weibull baseline hazard rate with parameters *κ* = 0.5 (left panel), λ = *κ* = 1.0 (middle panel), and *κ* = 1.5 (right panel).

**Figure 5:**
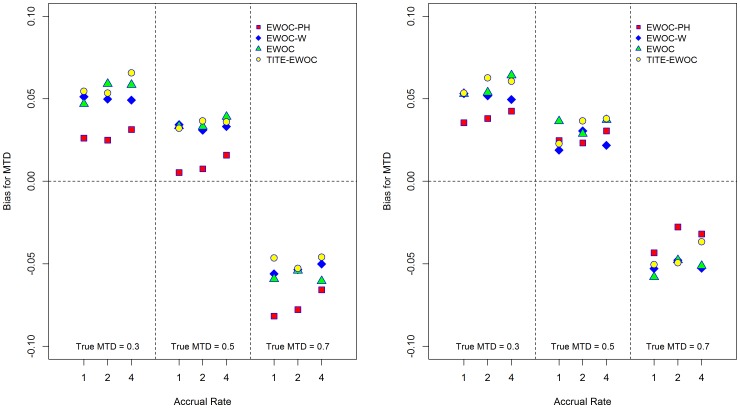
Average bias of the estimated MTD for each of the four models and nine scenarios when the time to DLT is generated from a non-proportional hazards model. *β*_2_ = 0.5 (left panel), *β*_2_ = 2.0 (right panel).

**Figure 6:**
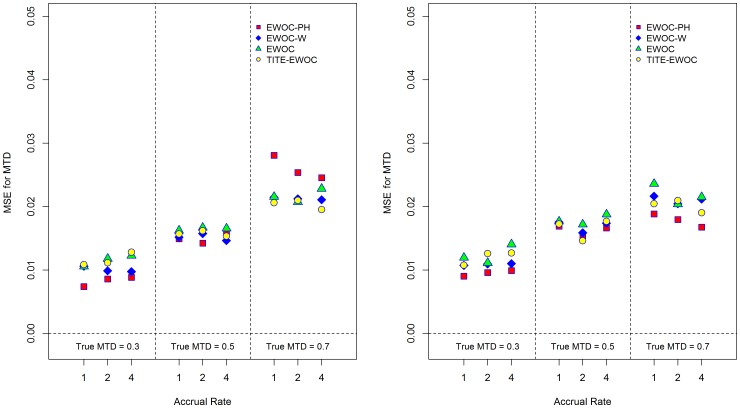
Mean square error of the estimated MTD for each of the four models and nine scenarios when the time to DLT is generated from a non-proportional hazards model. *β*_2_ = 0.5 (left panel), *β*_2_ = 2.0 (right panel).

Figure 3 shows that EWOC-PH has smaller average bias relative to TITE-EWOC and the other two versions of EWOC under all scenarios when *κ* = 0.5, 1. When *κ* = 1.5, EWOC-PH has a larger bias relative to TITE-EWOC but the extent of this difference is much smaller compared to the differences observed when *κ* = 0.5. The MSE for all models shown in Figure 4 are fairly close in most scenarios. Similarly, Figure 5 shows that when *β*_2_ = 0.5, the absolute average bias for EWOC-PH is smaller than the average bias using the other 3 models except when the true MTD is high (*γ* = 0.7). When *β*_2_ = 2.0, EWOC-PH has smaller absolute average bias relative to TITE-EWOC in 8 out of the 9 scenarios. In the 9^th^ scenario corresponding to the true MTD *γ* = 0.5 and accrual rate *ν* = 1, EWOC-PH and TITE-EWOC have about the same bias. Again, the MSE for all models shown in Figure 6 are very close in most scenarios. Based on these results, EWOC-PH does better than TITE-EWOC in the majority of these scenarios and model misspecifications combined. These cases occur when the cdf of the model from which the time to DLT is generated is above the cdf of the true model when the dose *x* is below the true MTD, see Figures 1 and 2.

**Figure 7:**
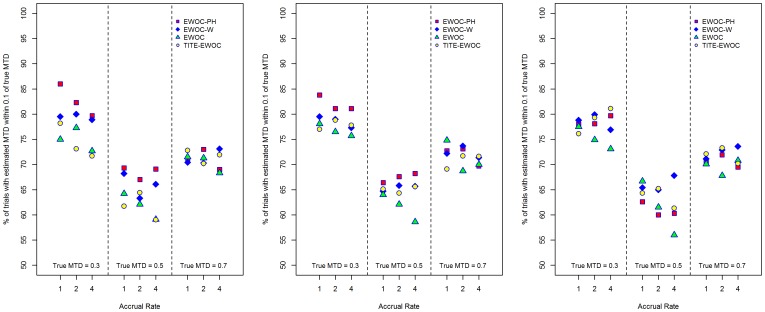
Percent of trials with estimated MTD within 0.1 of *γ*_true_ for each of the four models and nine scenarios when the time to DLT is generated from a proportional hazards model with Weibull baseline hazard rate with parameters *κ* = 0.5 (left panel), *κ* = 1.0 (middle panel), and *κ* = 1.5 (right panel).

**Figure 8:**
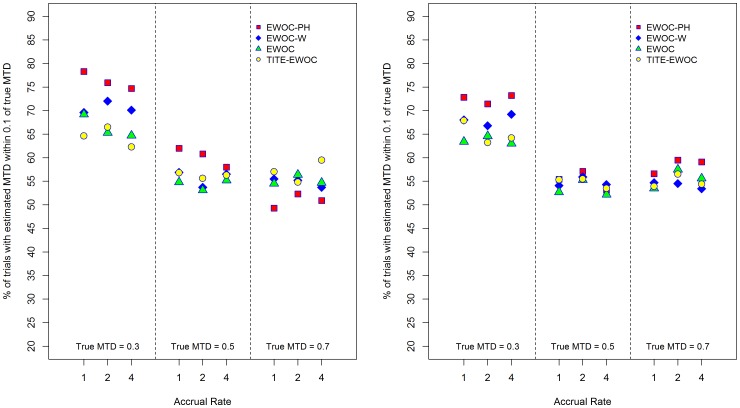
Percent of trials with estimated MTD within 0.1 of *γ*_true_ for each of the four models and nine scenarios when the time to DLT is generated from a non-proportional hazards model. *β*_2_ = 0.5 (left panel), *β*_2_ = 2.0 (right panel).

Figure 7 shows that EWOC-PH does better than the other models in terms of percent of trials with estimated MTD within 0.1 of the true MTD in the majority of the scenarios when *κ* = 0.5, 1. The extent of this difference can be as high as 12% between EWOC-PH and EWOC. This occurs when the true MTD is low (*γ* = 0.3) and the accrual rate is *ν* = 1. When *κ* = 1.5, the results are mixed and depend on the value of the true MTD and accrual rate. However, in eight out of the nine scenarios, the percent of trials with estimated MTD within 0.1 of the true MTD for EWOC-PH and TITE-EWOC are very close. When the time to DLT is generated by a non-proportional hazards model, Figure 8 shows that when *β*_2_ = 0.5, the percent of trials with estimated MTD within 0.1 of the true MTD using EWOC-PH is higher than the corresponding percentages using the other 3 models in six out of the nine scenarios. TITE-EWOC does better when the MTD is high, *γ* = 0.7. Similarly, when *β*_2_ = 2.0, the percent of trials with estimated MTD within 0.1 of the true MTD using EWOC-PH is higher than the corresponding percentages using the other 3 models in seven out of the nine scenarios. These percentages are very close in the case where the true MTD is *γ* = 0.5. Based on these summary statistics, we conclude that EWOC-PH is a good alternative design to TITE-EWOC since it may result in a smaller bias under the majority of scenarios considered here and some model misspecification.

### 4.3 Trial Safety

**Figure 9:**
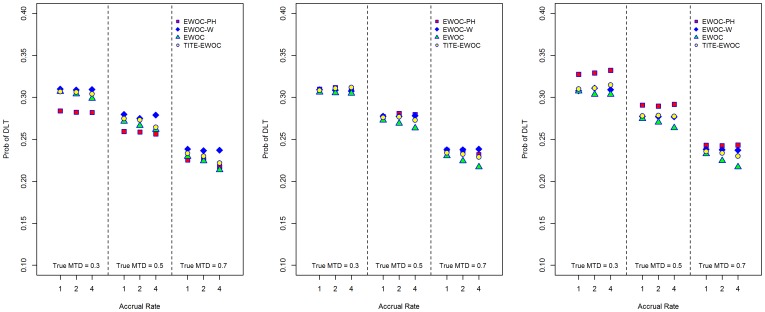
Average proportion of DLTs for each of the four models and nine scenarios when the time to DLT is generated from a proportional hazards model with Weibull baseline hazard rate with parameters *κ* = 0.5 (left panel), *κ* = 1.0 (middle panel), and *κ* = 1.5 (right panel).

**Figure 10:**
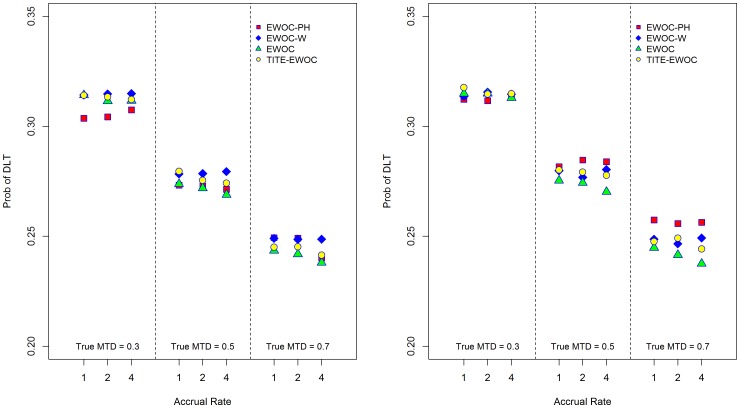
Average proportion of DLTs for each of the four models and nine scenarios when the time to DLT is generated from a non-proportional hazards model. *β*_2_ = 0.5 (left panel), *β*_2_ = 2.0 (right panel).

**Figure 11:**
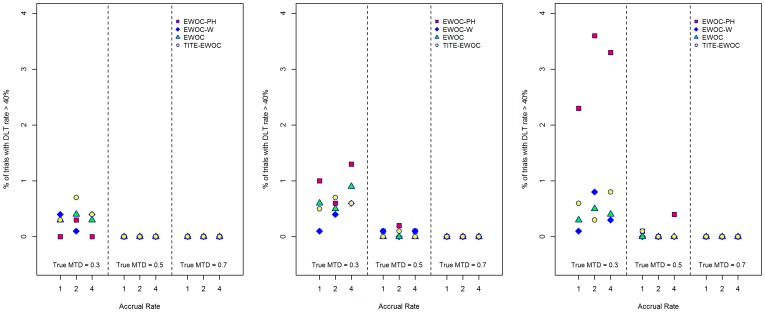
Percent of trials with DLT rate exceeding 40% for each of the four models and nine scenarios when the time to DLT is generated from a proportional hazards model with Weibull baseline hazard rate with parameters *κ* = 0.5 (left panel), *κ* = 1.0 (middle panel), and *κ* = 1.5 (right panel).

**Figure 12:**
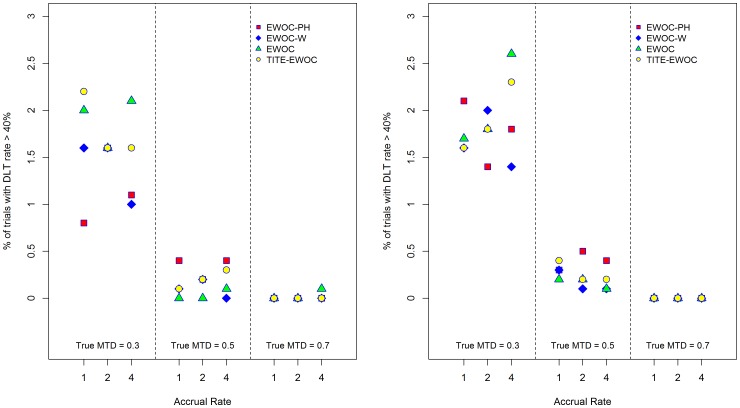
Percent of trials with DLT rate exceeding 40% for each of the four models and nine scenarios when the time to DLT is generated from a non-proportional hazards model. *β*_2_ = 0.5 (left panel), ***β***_2_ = 2.0 (right panel).

Figures 9 and 10 show that the average proportion of patients exhibiting DLT does not exceed *θ* = 0.33 using all four models for all scenarios and under the two model misspecifications. Furthermore, Figures 11 and 12 show that the estimated probability that a prospective trial will result in an excessive number of DLTs, defined as a DLT rate exceeding 40%, is very small and does not exceed 0.04 under all 9 scenarios and all the different models for generating the time to DLT considered here. We conclude that in general, designing a trial using EWOC-PH is safe but additional *ad hoc* stopping rules for excessive toxicity should always be put in place in writing the protocol.

### 4.4 Model Robustness

For a given scenario, Figures 3 and 5 show that the largest difference in the average bias using EWOC-PH is around 0.12, or 12% of the dose range. This occurs when the true MTD *γ* = 0.7, accrual rate *ν* = 1, and *κ* = 1.5 (Figure 3) and the true MTD *γ* = 0.7, accrual rate *ν* = 1, and *β*_2_ = 2.0 (Figure 5). When the true MTD is *γ* = 0.3, the largest difference in the average bias using EWOC-PH under the Weibull hazards corresponding to *κ* = 0.5, 1.0, 1.5 and the non-proportional hazards models corresponding to *β*_2_ = 0.5, 2.0 is about 0.03. When the true MTD is *γ* = 0.5, the largest difference in these average biases is around 0.065. Similarly, when comparing the percent of trials with estimated MTD within 0.1 of the true MTD for a given scenario, Figures 7 and 8 show that the largest differences between these percentages across the different models for generating the times to DLT is around 10%. We conclude that EWOC-PH is reasonably robust in terms of precision of the estimate of the MTD under the model misspecifications considered here.

Figures 9 and 10 also show that EWOC-PH is robust under the model misspecifications considered here where the largest difference in the average probability of DLT is about 0.05. This occurs when the true MTD is *γ* = 0.3 and the times to DLT are generated from a proportional hazards model with Weibull baseline hazards corresponding to *κ* = 0.5 and *κ* = 1.5. Similarly, the largest difference in the percent of trials with DLT rate exceeding 40% is 0.036. Again, this occurs under the same scenario and models misspecification discussed above, see Figure 11.

### 4.5 Real Trial Example

We designed a cancer phase I clinical trial in order to estimate the MTD of Veliparib (ABT-888) in combination with fixed doses of Gemcitabine and Intensity Modulated Radiation Therapy in patients with locally advanced, un-resectable pancreatic cancer. The trial has just been approved by the FDA and the first patient was enrolled at the time of writing of the manuscript. The MTD is defined to be the dose level of Veliparib that when administered to a patient results in a probability equal to *θ* = 0.4 that a dose limiting toxicity will be manifest within ten weeks, *τ* = 10. Due to the long length of the observation window to resolve DLT status, EWOC-PH was used to design the trial.

The dose for the first patient in the trial was set at 20 mg, previous results indicating this to be a safe dose. The dose for each subsequent patient will be determined so that, on the basis of all available data, the probability that it exceeds the MTD is equal to a pre-specified feasibility bound *α* = 0.25. The prior distribution of the MTD is based on the correlated priors model *M*4 described in Tighiouart et al. [13] where the support of the MTD is (0, ∞). After extensive consultation with the principal investigator (PI) of the trial, we will assume that the a priori probability that the MTD exceeds 100 mg is 10%. Upon completion of the trial, the MTD will be estimated as the median of the marginal posterior distribution of the MTD.

**Figure 13:**
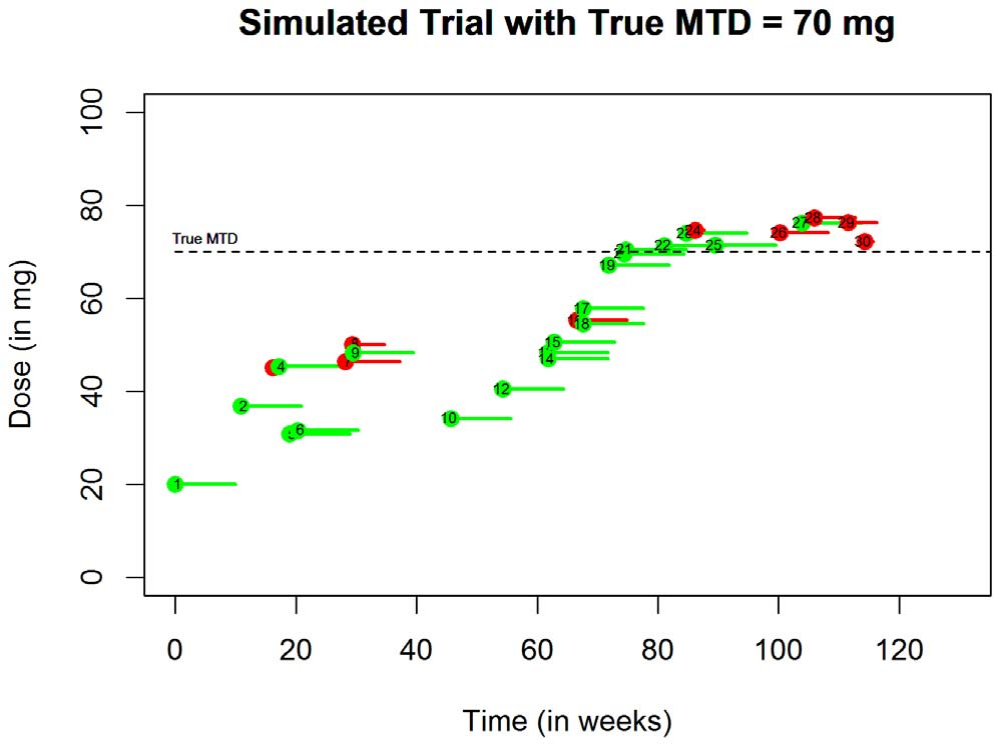
A simulated trial with true MTD  =  70 mg. The red pins indicate DLT and the size of the pins correspond to the time to DLT since patients are given the treatment. The green pins indicate that a patient did not experience DLT within one cycle of treatment.

Figure 13 shows an example of a simulated trial when the true value of the MTD *γ* = 70 mg and the probability of DLT at the initial dose *ρ_0_* is 0.05. Patients enter the trial according to a time homogeneous Poisson process with an average number of 3 patients per 10 weeks. This figure shows patients' number, the time when they enter the trial, the DLT status and how long it took to exhibit DLT if they did. To assess design operating characteristics, we simulated 1000 trials under 3 scenarios for the true value of the MTD *γ*. In each case, the probability of DLT at the initial dose is 0.05, the arrival times follow a time homogeneous Poisson process with rate equal to 3 patients per cycle, and *n* = 30 patients per trial. Table 2 shows the summary statistics based on 1000 trials. We can see that the estimated MTD is close to the true underlying *γ* and the overdose protection property of EWOC is illustrated by the last row of Table 2.

**Table 2:** Design operating characteristics based on 1000 trial replicates.

	True MTD		
	40	70	100
Estimated MTD	47	74	99.5
Percent of DLT	29	33.9	22.1

## Discussion and Conclusion

We proposed a Bayesian adaptive design for dose finding studies with the distinctive feature that it takes into account the time for a patient to exhibit DLT. The method is an extension of EWOC where the dose allocated to a patient is based on the doses allocated to previously and currently treated patients and the time it takes to exhibit DLT. The design is dynamic in the sense that patients can enter the trial at any time and the dose allocated to a patient makes use of all the information available at the time the patient enters the trial. We used a proportional hazards model with exponential baseline hazard function *h*_0_(*t*;*µ*) to describe the dose-toxicity relationship for simplicity. The assumption of constant baseline hazard function is reasonable since the parameter of interest *γ* depends on *h*_0_(*t*;*µ*) only through the cumulative baseline hazard *H*_0_(*t*;*µ*) in the observation window [0, *τ*] as shown in equation (2.5).

Simulation studies showed that EWOC-PH has smaller average bias when estimating the MTD compared to EWOC, TITE-EWOC, and EWOC-W in the majority of scenarios considered and under two different model misspecifications. Moreover, the estimated probability that a prospective trial will result in an estimate of the MTD within 10% of the dose range of the true MTD is higher when using EWOC-PH relative to EWOC, TITE-EWOC, and EWOC-W in the majority of scenarios and under model misspecification. We have also shown that EWOC-PH results in a safe trial design under all scenarios and model misspecification considered here. Furthermore, EWOC-PH is practically robust with respect to trial safety and efficiency under reasonable model misspecification. Therefore, we conclude that EWOC-PH is a good alternative design for late-onset toxicity cancer phase I trials relative to TITE-EWOC.

We applied this methodology to design a phase I cancer trial in which the length of the cycle is 10 weeks due to the use of radiation. In this trial, the PI could not provide an upper bound for the support of the MTD. However, based on previous clinical trials using ABT-888 in combination with Gemcitabine, the PI believes that the probability that the MTD exceeds 100 mg is about 10% a priori. Therefore, we used a class of correlated priors for *γ* and *ρ*_0_ described in [13]. To our knowledge, this is the first cancer phase I trial which uses time to DLT and an unbounded support for the MTD a priori.

An important characteristic of EWOC-PH is the effect of the length of time a patient is under observation on the dose recommended to the next patient. We have shown that the longer is a patient under observation with no evidence of DLT, the higher is the recommended dose for the next patient, assuming the DLT status of the previously treated patients has been resolved. This intuitively attractive property holds for TITE-EWOC and TITE-CRM as shown by Theorem 1. We have also shown that if a patient exhibits DLT shortly after treatment starts, then the recommended dose for the next patient is much lower than the recommended dose had the previous patient experienced DLT later on in the cycle. This property is not shared by either TITE-EWOC or TITE-CRM since patients given the same dose and who exhibit DLT at different time points contribute the same information to the likelihood function.

The design we presented is Bayesian and the dose allocated to the next patient corresponds to the estimate of the MTD *γ* having minimal risk with respect to the asymmetric loss function *l_α_*(*x*,*y*) =  *α* (*γ*−*x*) if *x*<*γ*, and *l_α_*(*x*,*y*) =  (1−*α*) (*x*−*γ*) otherwise. As such, it belongs to the “type I Best Intension design” defined by Fedorov et al. [27]. While the original EWOC [12] design is consistent in probability under a one parameter logistic model, we do not have a proof that this new design is consistent due to its added complexity. We did some simulations (data not shown) and we observed that the average bias of the estimate of the MTD decreases as a function of the sample size in the trial under the true model.

The methodology presented here assumes continuous dose levels of the agent under study, which is not uncommon in practice. When a prospective trial uses a pre-specified number of dose levels, we can apply this method by rounding down the dose recommended by EWOC-PH to the nearest available dose in the trial as proposed in [11], [17]. The corresponding design will likely suffer the drawbacks of large variability of the distribution of the number of cohorts treated at the MTD relative to the “Up-and-Down” designs, see Oron and Hoff [28]. However, it is important to note that the use of “Up-and-Down” designs is impractical in this setting due to the fact that the length of a cycle of therapy is much longer than the traditional 3 or 4 weeks follow up and the status of DLT must be resolved before enrolling the next cohort of patients. For instance, Table 1 shows that with an accrual rate of 2 patients per month, the median duration of a trial using EWOC-W (or an “Up-and-Down”) design is 64.5 months relative to 25.2 months for EWOC-PH, which is practically not feasible.

We plan to make available a software application for trial design and operating characteristics evaluation of cancer phase I trials using EWOC-PH. This would be an addition to our established Web based application of EWOC [29]. We are also working on adapting EWOC-PH to account for patients' baseline characteristics as in [15] and extending the binary DLT outcome to an ordinal toxicity grade, see [16] for an extension of EWOC to account for an intermediate grade of toxicity. Since our model makes use of the proportional hazards assumption, design operating characteristics under model miss-specification should always be studied under practical scenarios when designing prospective trials using this design. At the end of the trial, if there is evidence that the proportional hazards assumption is violated, standard techniques including the use of time varying covariate [30] will be carried out to analyze the data. Finally, extending EWOC-PH for determining the MTD curve of drug combination of two agents is under investigation.
